# Genome Editing Reveals Idiosyncrasy of CNGA2 Ion Channel-Directed Antibody Immunoreactivity Toward Oxytocin

**DOI:** 10.3389/fcell.2018.00117

**Published:** 2018-09-20

**Authors:** Janna Blechman, Savani Anbalagan, Gary G. Matthews, Gil Levkowitz

**Affiliations:** ^1^Department of Molecular Cell Biology, Weizmann Institute of Science, Rehovot, Israel; ^2^Department of Neurobiology and Behavior, Stony Brook University, New York, NY, United States

**Keywords:** neuropeptide, cGMP-gated ion channel, neurohypophysis, monoclonal antibody, oxytocin

## Abstract

Presynaptic cGMP-gated ion (CNG) channels positively or negatively modulate neurotransmitter secretion as well as the strength of synaptic transmission. Zebrafish cGMP-gated ion channel, CNGA2a (a.k.a. CNGA5), was previously reported to be specifically enriched in synaptic terminals of zebrafish oxytocin (OXT) neurons. This conclusion was based on immunoreactivity of a monoclonal antibody (mAb) clone L55/54, which was directed against the carboxy terminal tail of the CNGA2a. To study the role of CNGA2a in oxytocin neurons function, we generated zebrafish mutants of *cnga2a, cnga2b* and *oxt* genes using clustered regularly interspaced short palindromic repeats (CRISPR)-mediated genome editing. We show that mAb L55/54 specifically recognizes CNGA2a protein when expressed in heterologous cell culture system. Surprisingly, anti-CNGA2a immunoreactivity was not eliminated following knockout of either *cnga2a, cnga2b* or both. However, knockout of *oxt* resulted in total loss of anti-CNGA2a mAb immunoreactivity despite the lack of sequence and structural similarities between OXT and CNGA2a proteins. Our results provide a noteworthy lesson of differences in antibody immunoreactivity, which could only be revealed using specific genetic tools.

## Introduction

The hypothalamo-neurohypophyseal system (HNS) is an important neuroendocrine structure that coordinates brain neuronal circuits with peripheral organs responses to maintain body homeostasis (Gutnick and Levkowitz, 2012). The structure and function of the HNS is conserved in all vertebrates. It is composed of hypothalamic neurosecretory cells that produce the cyclic nonapeptides oxytocin (OXT) and arginine vasopressin (AVP). These peptides are packed into large dense-core vesicles, transported along axons that terminate in the posterior pituitary lobe (neurohypophysis) and are released to influence the function of target cells throughout the body (Burbach et al., 2001; Engelmann and Ludwig, 2004; Knobloch and Grinevich, 2014; Wircer et al., 2017). In addition to acting as neuroendocrine hormones, both OXT and AVP also act as neurotransmitters in the central nervous system (CNS) where they modulate social affiliation, stress, learning, and memory functions (Murphy et al., 2012). Notably, both neuroendocrine and CNS functions of OXT and AVP are conserved in evolution (Wircer et al., 2016). Given the central roles of OXT and AVP in animal physiology, deciphering the mechanisms underlying their neurosecretion has been the subject of many studies for over 60 years (Leng et al., 2015).

CNGA5 protein belongs to a family of cyclic-nucleotide gated (CNG) channels representing a family of cation channels shown to mediate cAMP and cGMP signaling in sensory neurons (Kaupp and Seifert, 2002; Podda and Grassi, 2014). CNG channels play roles in a number of activity-dependent modulatory and adaptive changes in neurons, in the regulation of a voltage-independent mode of Ca^2+^ entry, modulation of neurotransmitter release from presynaptic terminals and modification of synaptic strength (Podda and Grassi, 2014). CNG channels were shown to modulate transmitter release in retinal cone synapses and in the olfactory bulb as well as GnRH neuropeptide release in the hypothalamus (Bradley et al., 1997; Vitalis et al., 2000; Charles et al., 2001; El-Majdoubi and Weiner, 2002). Surprisingly, it was reported that a zebrafish CNG subunit, denoted CNGA5, exhibits restricted brain-specific expression pattern with only weak expression in the olfactory bulb (Tetreault et al., 2006). A subsequent study by Khan et al. (2010) employed a monoclonal anti-CNGA5 antibody and concluded that CNGA5 protein is enriched in synaptic terminals of zebrafish OXT neurons. This inspired us to examine the role CNGA5, which was recently renamed CNGA2a, in the modulation of OXT presynaptic activity.

To study the role of CNGA5 in the regulation of OXT function, we first performed analysis of the above-mentioned anti-CNGA5 L55/54 mAb immunoreactivity in larval and adult zebrafish in combination with genetic ablation of *cnga2a/b* and *oxt* genes. This analysis revealed unexpected discrepancy between *in vitro* and *in vivo* antibody reactivity that could only be shown using specific genetic tools.

## Results

### Anti-CNGA2a Antibody Immunoreactivity *in vivo* and in Heterologous Cell Culture

**FIGURE 1:**
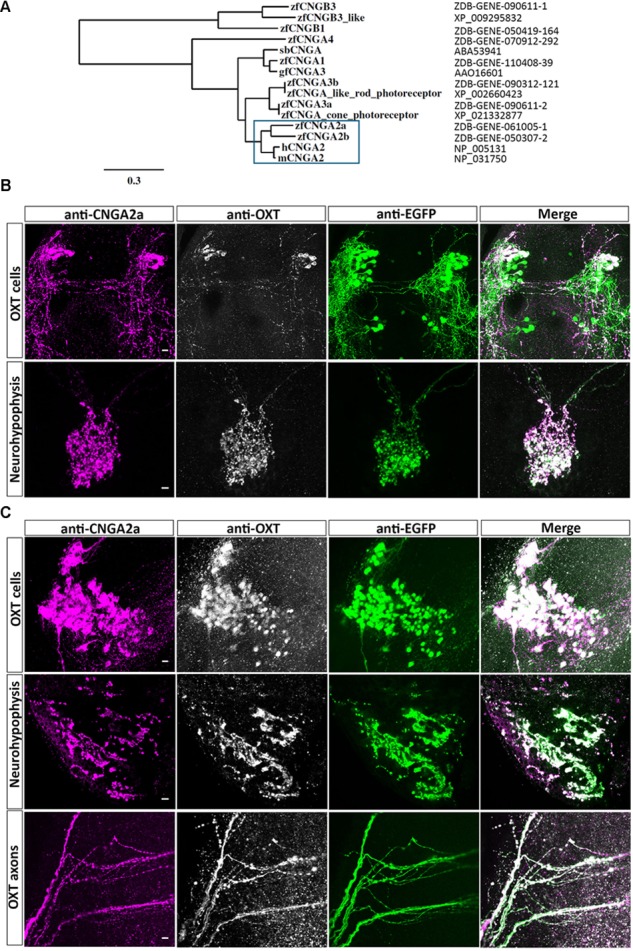
Immunoreactivity of anti-CNGA2a mAb in larval and adult zebrafish. **(A)** Phylogram of the CNG channels protein sequences. Comparison of the CNGA2 protein homologues from zebrafish, human, and mouse species is enclosed in the blue box. The scale bar indicates 30% amino acid residues substitution. **(B,C)** Confocal images showing representative labeling of the OXT perikarya, neuronal projections, and neurohypophyseal axonal termini with anti-CNGA2a mAb (magenta) and anti-OXT Ab (gray scale) in the context of the EGFP-positive OXT-ergic population in *oxt:egfp* reporter (green). Immunohistochemical analysis show colocalization of EGFP+, OXT+, and CNGA2a+ moieties in the cell bodies, axons, and nerve termini in the neurohypophysis of 6-day-old larva (*n* = 30/30) **(B)** and dissected brain and pituitary from 3-month-old adult zebrafish (*n* = 3/3) **(C)**. Scale bars: 10 μm.

Three mammalian cGMP-gated ion channel alpha subunits (CNGA), termed, CNGA1, CNGA2, and CNGA3 are widely expressed in the brain and play roles in visual and olfactory receptor neurons (Podda and Grassi, 2014). Tetreault et al. (2006) reported that a novel CNG isoform, which they named CNGA5, is specifically expressed in the brain. We performed phylogenetic analysis of zebrafish CNGA proteins using the current zebrafish genome database (GRCz10/danRer10) and Phylogeny.fr software (Dereeper et al., 2008). This analysis indicated that the previously named zebrafish *cnga5* (ZFIN ID: ZDB-GENE-061005-1) and *cnga2* (ZFIN ID: ZDB-GENE-050307-2) genes are both close homologs to human and mouse *CNGA*2 genes (**Figure 1A** and **Supplementary Figure S1**). We therefore suggest that zebrafish CNGA5 is a paralog of CNGA2, which arose from the ancient ray-finned fish genome duplication. In concurrence with the nomenclature committee of the Zebrafish Information Network (ZFIN), we re-named the *cnga5* gene as *cnga2a* (ZFIN ID: ZDB-GENE-061005-1) and *cnga2* (ZFIN ID: ZDB-GENE-050307-2) as *cnga2b*.

Khan et al. (2010) generated a monoclonal antibody (mAb L55/54) which was raised against CNGA5/CNGA2a carboxy terminal tail. Based on the immunoreactivity of this antibody they concluded that CNGA2a is enriched in synaptic terminals of zebrafish OXT neurons. Using the zebrafish transgenic OXT reporter line, Tg(*oxt*:EGFP) (Blechman et al., 2011; Gutnick et al., 2011), we were able to confirm that mAb L55/54 display strong immunoreactivity which co-localized with anti-OXT immunofluorescence in 6-day-old EGFP-labeled larval hypothalamic neurons and their hypothalamo-neurohypophyseal axonal termini (**Figure 1B**). Similarly, colocalization of mAb L55/54 and anti-OXT immunoreactivity was observed in hypothalamic neuronal perikarya, projecting axons, and neurohypophyseal termini of the adult Tg(*oxt*:EGFP) zebrafish (**Figure 1C**). However, despite the above results we failed to detect *cnga2a* and *cnga2b* mRNA in zebrafish OXT neurons by *in situ* hybridization (**Supplementary Figure S2**). Notably, lack of antibody staining of OXT in the posterior EGFP-labeled OXT neuronal cluster (**Figure 1B**) is in line with our previous published findings (Wircer et al., 2017).

**FIGURE 2:**
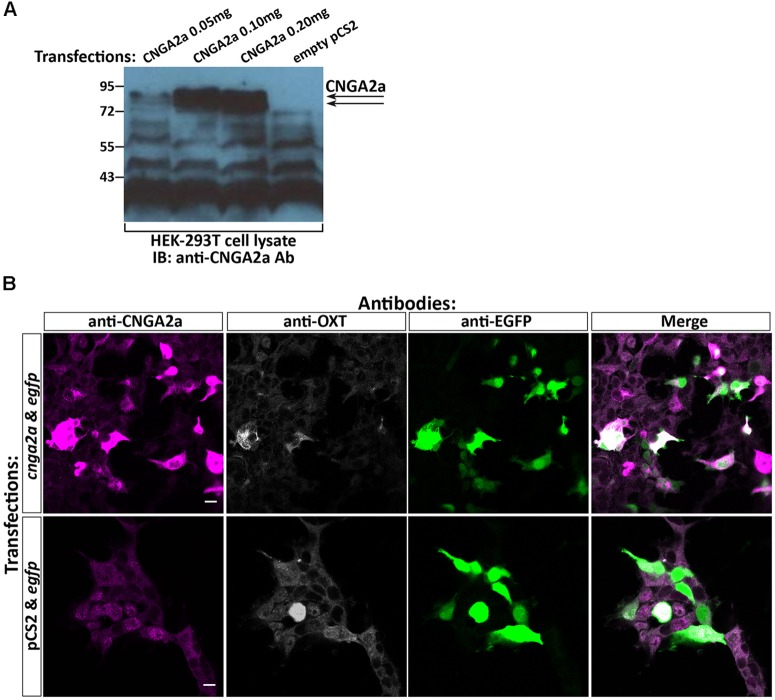
Specificity of anti-CNGA2a mAb in heterologous expression system. **(A)** Western blot analysis of HEK293T cells transfected with *cnga2a* cDNA. HEK293T cells were transiently transfected with different amounts of *cnga2a* cDNA or a mock plasmid and were harvested 48 h post-transfection. Western blot analysis of equal amounts of protein extracts were performed using anti-CNGA2a mAb. The correct position of the doublet CNGA2a protein bands are marked by arrowheads (*n* = 2/2). **(B)** Confocal images of HEK293T cells transfected with *cnga2a* cDNA. HEK293T cells were transiently co-transfected with combinations of *egfp* cDNA either with *cnga2a* cDNA or an empty pcS2 plasmid. Forty-eight hours post-transfection the monolayer cultures were fixed in 3% paraformaldehyde (PFA), permeabilized with 0.5% Triton-X100/3% PFA, washed in PBS and fluorescently co-stained with anti-CNGA2a (magenta), anti-OXT (gray scale), and anti-GFP (green) antibodies (*n* = 4/4). Scale bars: 10 μm.

To confirm that mAb L55/54 recognizes the CNGA2a epitope we have transiently expressed the full-length *cnga2a* cDNA in HEK293T cell line and performed Western blot analysis. In this assay, the mAb L55/54 antibody detected two protein bands with an apparent molecular weight of around 72–80 kDa in *cnga2a*-transfected cell lysates but not in the empty vector-transfected control (**Figure 2A**). These protein bands corresponded to the expected molecular weight of the presumably glycosylated zebrafish CNGA2a protein. Additional lower molecular weights protein bands appearing in all transfections, including the vector alone-transfected cell lysate, suggested possible cross-reactivity to other proteins. mAb L55/54 also displayed positive immunofluorescent staining (IFS) of *cnga2a*-transfected HEK293T monolayer cell culture but not of control cells that were co-transfected with empty vector and EGFP expression plasmids (**Figure 2B**).

We conclude that the CNGA2a-directed mAb recognizes CNGA2a protein *in vitro*, when the latter is expressed in heterologous cell culture.

### Knockout and Knockdown of CNGA2 Orthologs Do Not Affect Anti-CNGA2a Immunoreactivity

**FIGURE 3:**
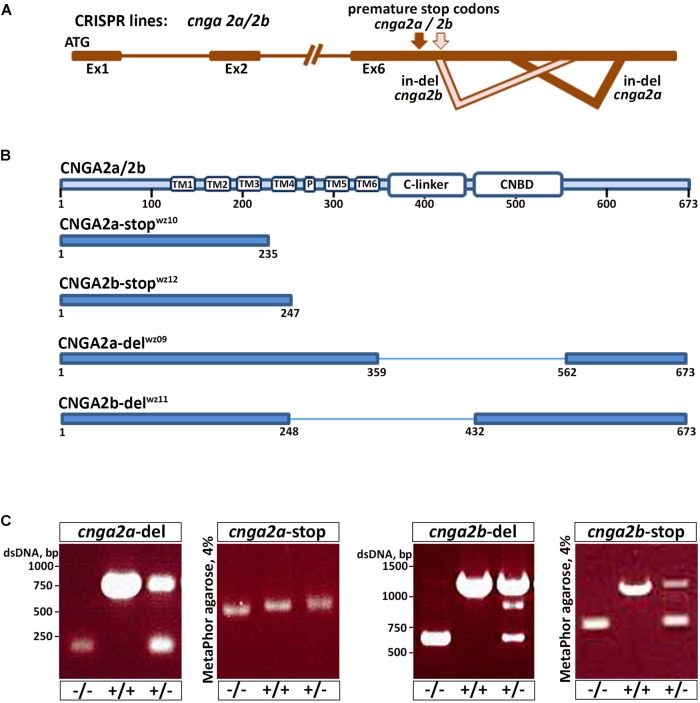
*cnga2* isoforms genome editing using CRISPR/Cas9. **(A)** Schematic representation of the genetic structure of *cnga2* isoforms. *cnga2a* and *cnga2b*. **(B)** Schematic representation of the predicted CNGA2a and CNGA2b protein products following sequence analysis of germline transmitted CRISPR-induced mutation alleles. **(C)** Embryos and adult fish were screened by PCR for germline transmission using gene-specific primers. PCR products were resolved in 1% LE agarose gels for *cnga2a-*del and *cnga2b-*del progenies and in 4% MetaPhor gel for *cnga2a-*stop and *cnga2b-*stop progenies.

The highly localized immunoreactivity of anti-CNGA2a in OXT neurons and their projecting axons prompted us to examine the role of CNGA2a in the functionality of OXT neurons. We therefore employed the CRISPR/Cas9 gene targeting method to generate germline-transmitted mutant zebrafish lines of *cnga2a* and its paralogous gene *cnga2b*. For each gene, we have generated two types of mutant alleles (**Figure 3A**). Thus, CRISPR-mediated *indel* of 2 bp in *cnga2a* gene resulted in a nonsense mutation leading to the premature stop codon, we termed *cnga2a*-stop (**Figures 3B,C** and **Supplementary Figure S3B**). In a similar manner, we generated 17 bp *indel* mutation in *cnga2b* gene, which should lead to a truncated protein at amino acid residue 247 (*cnga2b-stop*) (**Figures 3B,C** and **Supplementary Figure S3D**). In addition, we used double-guided CRISPR strategy to generate two large in-frame deletions in exon6 of *cnga2a* and *cnga2b* encoding to amino acids 360–561 (*cnga2a-del*) and 249–431 (*cnga2b-del*), respectively. These large deletions abolish known functional domains attributed to CNG channels (**Figures 3B,C** and **Supplementary Figures S3A,C**).

**FIGURE 4:**
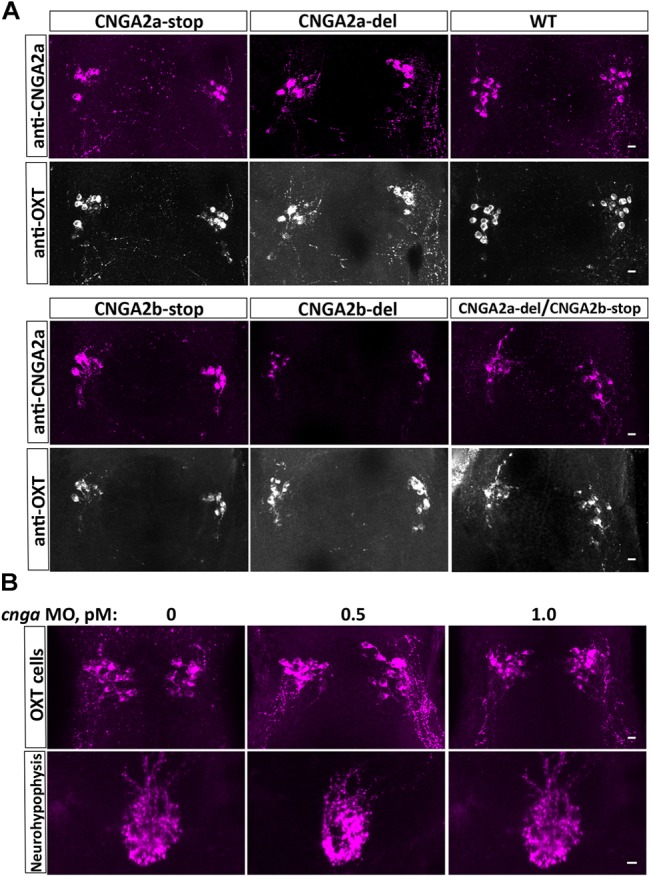
Anti-CNGA2a immunoreactivity is not affected following KO of *cnga2a* and/or *cnga2b*. **(A)** Immunostaining and confocal imaging of 6-day-old larva NPO of the wild type zebrafish (*n* = 30/30), *cnga2a* (*n* = 20/20), *cnga2b* (*n* = 16/16), and double *cnga2a/2b* (*n* = 6/6) zebrafish mutant variants with anti-CNGA (magenta) and anti-OXT (gray scale) antibodies. **(B)** Anti-CNGA2a mAb immunostaining and confocal imaging of the hypothalamus and neurohypophysis of 6-day-old larva injected with different concentrations of *cnga2a* missense morpholino oligonucleotide (*n* = 10/10). Scale bars: 10 μm.

Because the mAb L55/54 we used is directed to a 106 amino acids peptide that corresponds to the cytoplasmic C-terminal tail of CNGA2a (Khan et al., 2010) we expected reduced or no immunoreactivity in *cnga2a* mutants. Surprisingly, both *cnga2a-stop* and *cnga2a-del* mutant alleles retained mAb L55/5 immunoreactivity (**Figure 4A**). Similarly, knockdown of *cnga2a* by injecting antisense morpholino oligonucleotide (MO) had no effect on mAb L55/54 immunoreactivity (**Figure 4B**). To exclude the possibility that the retained anti-CNGA2a is due to cross-reactivity with CNGA2b paralog we also generated *cnga2a/b* double homozygous mutant (CNGA2a-del/CNGA2b-stop) and demonstrated that it still retained mAb L55/54 immunoreactivity (**Figure 4A**). These results suggested that mAb L55/54 immunoreactivity, which was detected in zebrafish OXT neurons by Khan et al. (2010), is due to an antigenic moiety that is different from the CNGA2a protein.

### Anti-CNGA2a Immunoreactivity Is Lost in OXT Knockout Zebrafish

**FIGURE 5:**
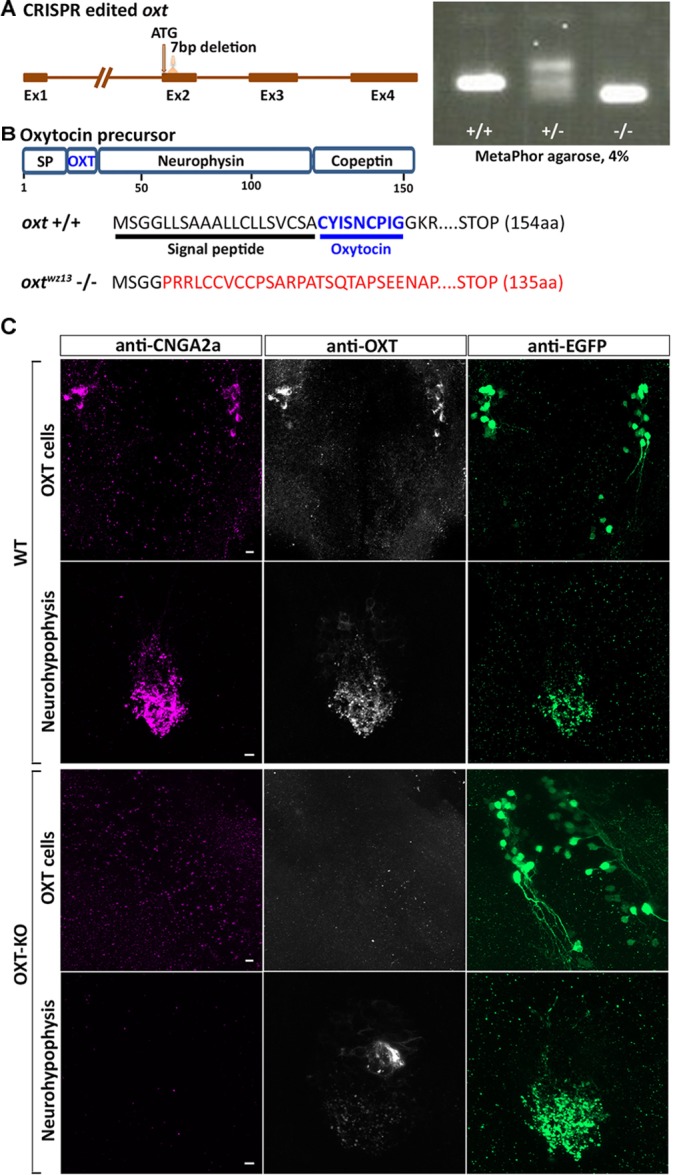
Anti-CNGA2a immunoreactivity is lost following KO of oxytocin. **(A)** Schematic representation of oxytocin (*oxt*) gene. OXT translation start site and sgRNA target site are both indicated with arrows. Embryos and adult fish were screened by PCR for germline transmission using gene-specific primers following PCR products separation in 4% MetaPhor gels. DNA sequence of mutant allele transmitted through the germline contained a 7 bp deletion. **(B)** Schematic representation of predicted translated products from *oxt* +/+ and *oxt* –/– alleles. **(C)** Confocal images showing representative anti-CNGA2a (magenta) and anti-OXT (gray scale) labeling of OXT cells and their neurohypophyseal axonal termini of OXT-KO 6-day-old larva in the background of a transgenic Tg(*oxt:egfp-3′utr*) reporter (green) (*n* = 12/12). Scale bars: 10 μm.

The nearly complete overlap of mAb L55/54 and anti-OXT immunoreactivity in OXT-ergic perikarya and axons inspired us to examine whether mAb L55/54 detects the OXT neuropeptide. To this end, we generated CRISPR-mediated germline transmitting OXT mutant harboring a 7 bp deletion in the second exon of the zebrafish *oxt* gene (**Figure 5A** and **Supplementary Figure S3E**). This CRISPR-mediated indel mutant lead to a frameshift mutation, which is predicted to abolish the expression of the OXT neuropeptide (**Figures 5A,B**). Indeed, no anti-OXT immunoreactivity was detected in the brains and neurohypophysis of homozygous *oxt -/-* mutant fish (**Figure 5C**). In accordance with our hypothesis that the anti-CNGA2a mAb L55/54 binds to OXT neuropeptide, mAb L55/54 immunoreactivity was not detected in *oxt -/-* mutant fish (**Figure 5C**). To verify that the lack of immunoreactivity in the *oxt -/-* mutant was not as a result of OXT neuronal cell loss we performed anti-OXT and anti-CNGA2a staining of *oxt -/-* mutant which was crossed with the OXT transgenic reporter, Tg(*oxt*:EGFP-*oxt*3′*UTR*). This experiment showed that the loss of OXT expression in the *oxt -/-* mutant fish had no effect on EGFP-positive OXT neurons survival and/or their neurohypophyseal projecting axons (**Figure 5C**).

Taken together, our results showed that although mAb L55/54 detects the CNGA2a protein *in vitro*, it’s *in vivo* immunoreactivity is due to an unpredicted antibody cross-reactivity toward OXT neuropeptide.

## Discussion

The report of CNGA2a expression in OXT neurons is of great interest as cGMP-gated ion channels play important roles in the modulation of neuronal activity, including the regulation of voltage-independent Ca^2+^ entry, neurotransmitter release from presynaptic terminals and synaptic strength (Zufall et al., 1997; Podda and Grassi, 2014). Thus, the possible involvement of CNGA2a in the regulation of OXT function is intriguing. However, the suggested localization of CNGA2a protein in OXT synaptic termini is based on anti-CNGA2a immunoreactivity in OXT neurons. Here, we tested the *in vitro* and *in vivo* immunoreactivity of a mAb L55/54, which was directed to CNGA2a. We show that while anti-CNGA2a-directed mAb recognizes the correct epitope *in vitro*, it has a surprisingly strong *in vivo* cross-reactivity toward the structurally unrelated oxytocin neuropeptide.

Notably, we were able to confirm the previous findings of Khan et al. (2010) who reported that mAb L55/54 recognizes an antigenic moiety, which is expressed in OXT-ergic synaptic termini. Nevertheless, the use of genome editing tools that were not available at the time of the original publication revealed that mAb L55/54 immunoreactivity was not eliminated following knockout of zebrafish *cnga2a* and *cnga2b* genes. It is possible that the CRISPR-mediated *cnga2a* and *cnga2b* and in particular, the internal deletions retained residual antigenic activity. However, knockout of the oxytocin gene resulted in the total loss of immunoreactivity. Moreover, previous studies reported prominent expression of CNGA2 mRNA in the olfactory placode (Barth et al., 1996; Sato et al., 2005). However, both Khan et al. (2010) as well the present study show that mAb L54/55 exclusively stain oxytocin neurons but failed to detect mAb L54/55 immunoreactivity in the olfactory placode or other brain areas. Thus, the immunoreactivity of mAb L55/54 toward OXT neurons is likely due to recognition of the oxytocin neuropeptide.

Monoclonal antibodies mostly demonstrate monospecificity, to a definite protein epitope, however, the cases of cross-reactivity, i.e., recognition of unrelated protein compounds, have been reported (Valentino et al., 1985; Breiteneder and Mills, 2006; Van Regenmortel, 2014). Such cross-reactivity could be due to antibody recognition of either a stretch of continuous amino acid sequence that forms a linear epitope, or a conformational epitope, namely a folded secondary structure that resulted from proximity of distant amino acids (Wilson and Stanfield, 1994; Dall’antonia et al., 2014). We showed that anti-CNGA2a mAb reacts specifically with both the unfolded denatured CNGA2a protein observed in the Western blot analysis as well as with the presumably native protein when overexpressed in heterologous cell culture. However, we found no amino acid sequence identity or similarity between the OXT precursor polypeptide and the C-terminal antigen of CNGA2a, to which mAb L55/54 was directed (**Supplementary Figure S1**). This suggests that the specific mAb L55/54 antigenic sequence within oxytocin peptide or its precursor is not represented by a continuous amino acid segment. Another possibility explaining the observed cross-reactivity of anti-CNGA2a mAb with OXT could be the recognition of a mimotope in the OXT structure that shows limited or even no sequence similarity to the protein immunogen but could mimic the shape or charge of the CNGA2a immune epitope (Knittelfelder et al., 2009; Huang et al., 2014).

Our results raise the question of whether CNGA2a is expressed in OXT neurons. Loss of anti-CNGA2a immunoreactivity in OXT-KO zebrafish may be explained by OXT-dependent CNGA2a expression or stability. However, we failed to detect expression of *cnga2a/b* mRNA by *in situ* hybridization.

Taken together, our results provide a noteworthy lesson of differences in antibody immunoreactivity, which could only be revealed using specific genetic tools. We submit that the specificity of mAb immunoreactivity *in vivo* should always be controlled by the genetic deficiency of the target protein.

## Materials and Methods

### Animals and Antibodies

Zebrafish were raised and bred according to standard protocols. All experimental procedures were approved by the Weizmann Institutional Animal Care and Use Committee (IACUC). Zebrafish transgenic line Tg(*oxt*:EGFP) contains a 644 bp upstream region of *oxt* promoter (Blechman et al., 2011; Gutnick et al., 2011), Tg(*oxt*:EGFP-*oxt*3′*UTR*) contains the abovementioned *oxt* promoter region combined with 600 bp downstream region of *oxt* gene.

Guinea pig polyclonal antibody directed to the oxytocin peptide was purchased from Bachem (Bachem California, Torrance, CA, United States, Cat. T-5021.0050). Rabbit anti-EGFP (A-11122; Life technologies/Thermo Fisher, Waltham, MA, United States) was used to detect transgenic EGFP expression. Anti-CNGA5 mAb was a generous gift of Dr. J. Trimmer (UC Davis, United States). Secondary antibodies were purchased from Jackson ImmunoResearch Laboratories (West Grove, PA, United States).

### Genome Editing Using CRISPR

Cas9 protein was produced by the Weizmann Institute of Science Protein purification unit using the pET-28b-Cas9-His (Alex Schier Lab Plasmids, Addgene, Cambridge, MA, United States) as a template. CRISPR sgRNAs were designed using CRISPR direct design^1^ and are listed in the **Supplementary Table S1**. CRISPR protocol was performed as described in Gagnon et al. (2014). Oligonucleotide containing the T7 promoter sequence upstream of specific target sites was annealed with a constant oligonucleotide bearing Cas9 binding site. sgRNA were generated by *in vitro* transcription using a T7 RNA polymerase MEGA short script T7 kit (Life Technologies, United States) and purified using miRNeasy kit (Qiagen, Germantown, MD, United States). Cas9 protein (600 ng) and sgRNA (200–400 ng) were co-injected at the one-cell stage, and at least five pooled embryos were used to evaluate the genomic mutation of the targeted genes by PCR analysis. Deletion mutants of *cnga2a* and *cnga2b* were generated by using two sgRNAs that resulted in large genomic deletions in the genes. Germline transmitting zebrafish mutants were generated using sgRNAs directed to the *oxt, cnga2a*, and *cnga2b* genes (**Supplementary Table S1**).

### Genotyping

Embryos or fin-clips of adult fish were placed in PCR tubes, with 50 μl of lysis buffer (50 mM NaOH) and incubated at 95 °C for 30 min. The samples were then neutralized by the addition of 5 μl of 1 M Tris–HCl (pH 7.5) and 2 μl were taken for 25 μl of PCR mix. WT, heterozygous and homozygous *oxt*^delta7^ and *cnga2a/2b-stop* animals were identified by high-resolution analysis of PCR reaction using 4% MetaPhor agarose (Lonza, Rockland, ME, United States) gels. *cnga2a/2b-del* animals were identified by the analysis of PCR using 1.5% SeaKem LE agarose (Lonza, Rockland, ME, United States) gels. For genotyping primer sequences see **Supplementary Table S1**.

### Microinjection of Morpholinos

Antisense morpholino (MO) oligonucleotide directed to *cnga2a translation start site* (Gene Tools, LLC, Corvallis, OR, United States) was used as described previously (Blechman et al., 2007). The stock solution of the translation blocking MO (5′-AACAACAGTTGACAGGTCATCCTGC -3′) was prepared by dissolving in distilled water at 1 mM concentration and embryos were micro-injected with an amount of 1 or 2 ng/embryo at the one-cell stage and allowed to develop at 28.5°C.

### Transient Transfection, Immunoblot and Immunofluorescent Staining

For *cnga5* expression in cell culture the *cnga5* open reading frame was cloned into the pcDNA3 vector containing DYK-tag to generate pcDNA3-DYK-cnga5 plasmid. HEK293 cells were grown either on glass coverslips or directly in 12-well plates and were thereafter transfected (at 60% confluence) with a total amount of 1.0 μg/well of either the pcDNA3-*Dyk-cnga5* expression vector or control pcDNA3 together with pcDNA3-*egfp* expressing vector (Addgene, Cambridge, MA, United States) using a standard calcium phosphate transfection method. Cells were harvested 48 h post-transfection in 150 μl of hot SDS sample buffer and 15 μl of the crude protein extract was fractionated by 8% SDS-PAGE followed by immunoblotting with an affinity-purified anti-CNGA5 antibody. Fixation and immunofluorescent staining (IFS) of cell monolayers expressing CNGA5 and EGFP proteins was performed as described previously (Nakamura et al., 2000) using anti-CNGA5, anti-OXT, and anti-GFP primary antibodies.

### Immunofluorescent Staining of Zebrafish

Embryos were collected at 6 days post-fertilization and fixed in 4% PFA/PBS. Brains and pituitaries were dissected from 3-month-old zebrafish and subjected to fixation in 4% PFA/PBS. For IFS, PFA-fixed larvae and adult tissues were washed in PBS, dehydrated using methanol 100% and stored at -20°C overnight. IFS was performed according to the protocol described in the zebrafish brain atlas (**RRID**:SCR_000606)^2^. The rehydrated samples were blocked in 500 μL of blocking solution (PBS + 10% goat serum + 1% DMSO + 0.3% Triton X100) that was then replaced with 200 μL of fresh blocking solution with commercial primary antibodies at 1:200 concentrations or 2 μg/ml of anti-CNGA5 mAb and incubated overnight at 4°C. Samples were washed and treated with 200 μL of corresponding secondary antibodies in blocking solution at 1:200 concentrations overnight at 4°C. Then, samples were washed and transferred to 75% glycerol. Embryos were mounted ventrally after removal of the jaws. Adult dissected pituitaries and brain tissues were whole-mounted.

### Imaging

Images of fluorescently labeled samples were obtained by using Zeiss LSM 800 inverted confocal microscope with 488, 561, and 647 nm lasers and oil immersion X 40 lenses. Maximum intensity projection images of the whole Z-stacks or subset of Z-stacks were generated using the Zen software (Zeiss). Processing of multiple channel images (i.e., linear adjustments of brightness, contrast and levels) was performed on individual channels using Photoshop CS7 Extended (Adobe).

## Author Contributions

JB and GL designed the study and wrote the manuscript. JB generated constructs and CRISPR mutants, and performed immunostaining, cell culture assays, Western blot, and confocal imaging. SA contributed to the initial conceptualization of the study and optimized the CRISPR/Cas9 method and genotyping. GM provided the affinity-purified anti-CNGA5 antibody, and shared his unpublished zebrafish *cnga2/3/5* EST sequences and bioinformatic analysis.

## Conflict of Interest Statement

The authors declare that the research was conducted in the absence of any commercial or financial relationships that could be construed as a potential conflict of interest.

## Footnotes

1 http://crispr.dbcls.jp

2 http://zebrafishbrain.org/protocols.php
