# Supplementary material for: Genome Editing Reveals Idiosyncrasy of CNGA2 Ion Channel-Directed Antibody Immunoreactivity Toward Oxytocin
Source: Front Cell Dev Biol. 2018 Sep 20;6:117. doi: 10.3389/fcell.2018.00117 (PMC6158348; doi:10.3389/fcell.2018.00117)
Supplement: Supplementary file 1 [file Data_Sheet_1.pdf]

*Supplementary Material*

# Genome editing reveals idiosyncrasy of CNGA2 ion channel- directed antibody immunoreactivity towards oxytocin

**Janna Blechman[1*], Savani Anbalagan[1], Gary G. Matthews[2], Gil Levkowitz[1*]**

[1]Departments of Molecular Cell Biology, Weizmann Institute of Science, Rehovot, Israel.

[2]Department of Neurobiology and Behavior, Stony Brook University, New York, USA.

*** Correspondence:**

Corresponding Authors
J.B., janna.blechman@weizmann.ac.il and/or G.L. gil.levkowitz@weizmann.ac.il

## 1 Supplementary Figures and Tables

### 1.1 Supplementary Figures

See next page.

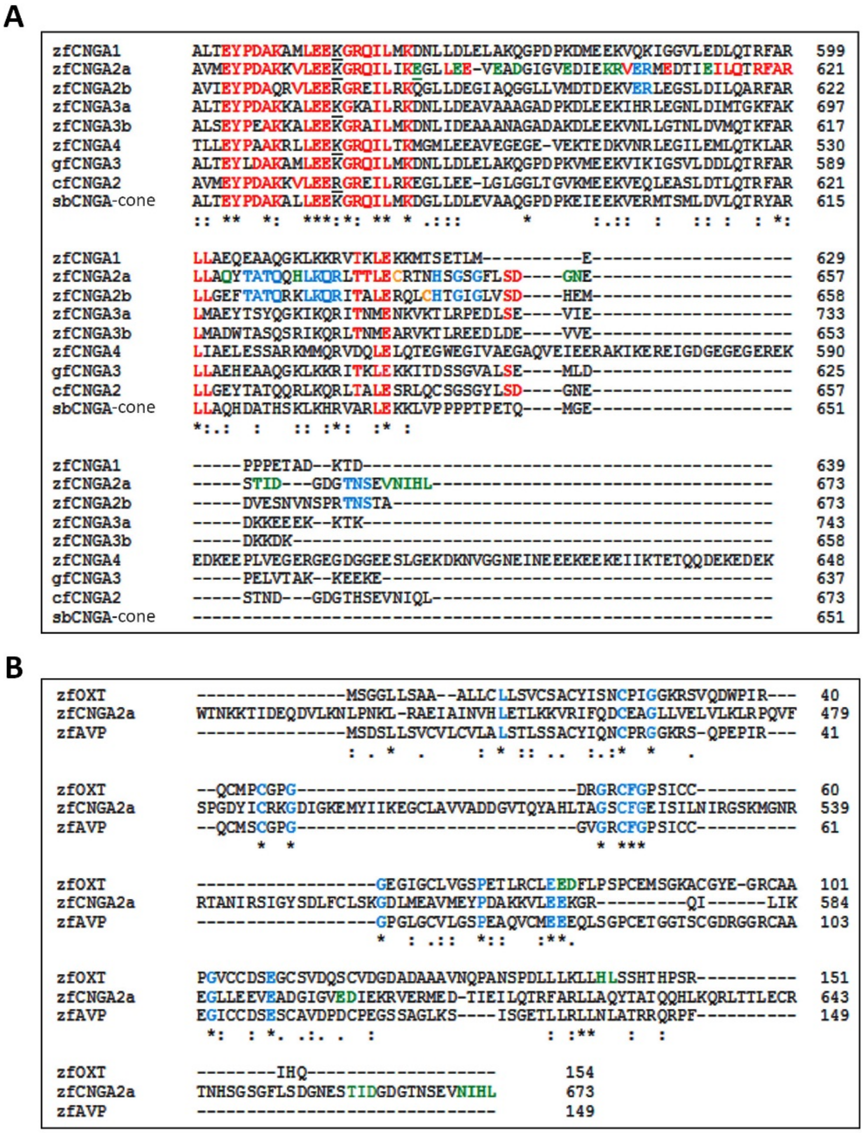


**Supplementary Figure 1. Analysis of potential CNGA2a recognition sites for anti-CNGA2a L55/54 mAb.**

(**A**) Alignment of C-terminal CNGA2a 106 amino acid epitope to the C-terminal sequences of the closely related CNGA channel protein sequences from the zebrafish and other species. Amino acid residues marked in red display conserved residues in all sequences. Identical residues between CNGA 2a and CNGA 2b proteins are marked in blue and green-colored letters indicate unique residues for CNGA2a but not CNGA2b. *zf*, zebrafish; *gf*, goldfish; *cf*, catfish; *sb*, striped bass.

(**B**) Alignment of CNGA2a C-terminal to oxytocin and arginine-vasopressin precursor protein sequences. Blue-colored letters indicate identical and structurally related amino acid residues that are common between all three protein sequences. Green letters mark common residues between CNGA2a and oxytocin but not arginine-vasopressin precursor sequences.

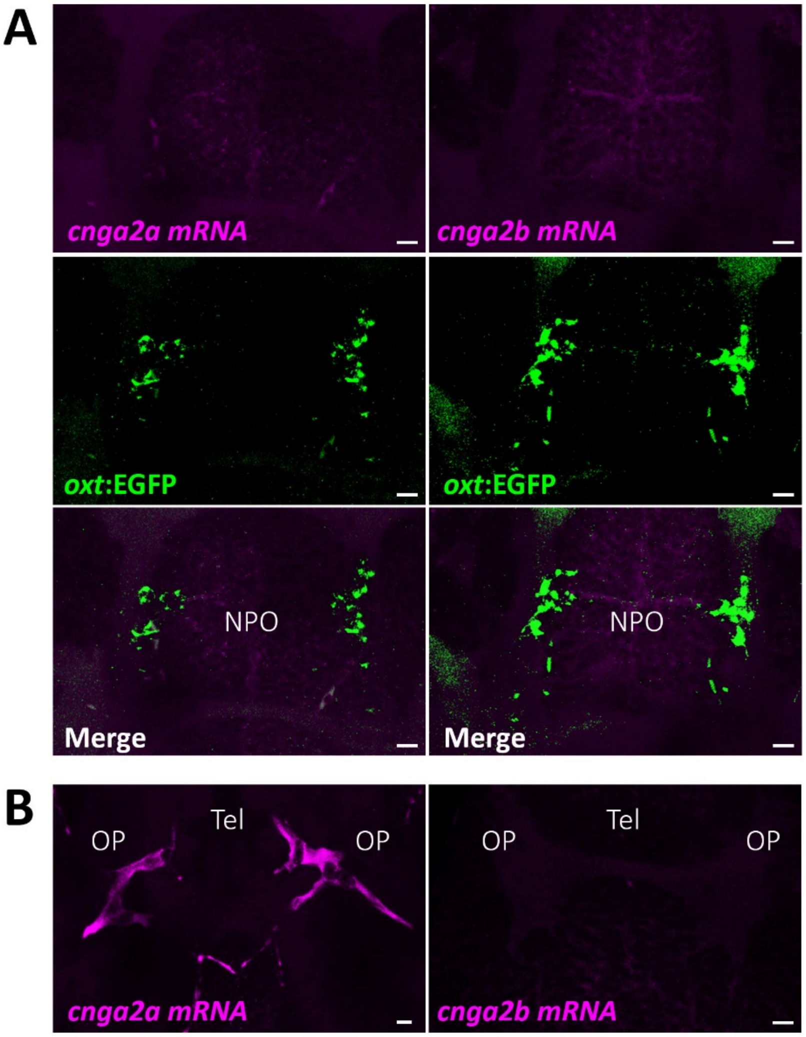


**Supplementary Figure 2. Expression of *cnga2a/b* mRNAs is not detected in zebrafish oxytocin neurons.**

(**A**) Confocal Z-stack images showing fluorescent in situ hybridization (FISH) of transgenic 6-dpf old Tg(*oxt*:EGFP) larvae using probes directed against mRNAs of *cnga2a* and *cnga2b*, followed by anti-EGFP staining. The NPO area with oxytocin cell bodies Tg(*oxt*:EGFP) are shown. No detectable expression of *cnga2a* and *cnga2b* is observed. Scale 10µm.

(**B**) *cnga2a* but not *cnga2b* is expressed in olfactory placade of 6dpf old larvae using probes directed against mRNAs of *cnga2a* and *cnga2b.* Scale 10µm.

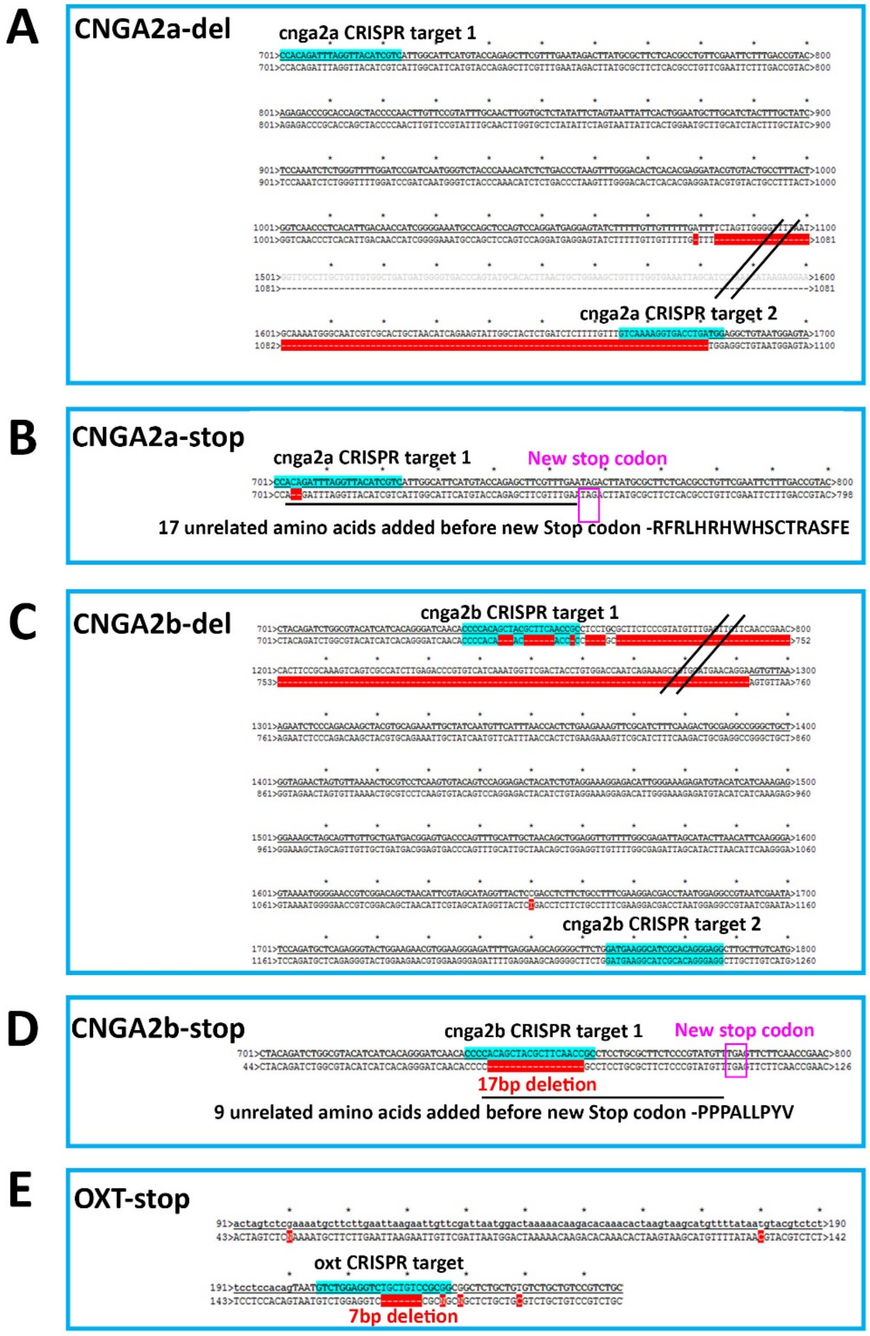


**Supplementary Figure 3. Analysis of *cnga2a* and *cnga2b* CRISPR-induced genomic mutations.**

Alignments of wild type and mutant alleles for *cnga2a*-del (**A**), *cnga2a*-stop (**B**), *cnga2b*-del (**C**), *cnga2b*-stop (**D**) and *oxt* (**E**) homozygous KO zebrafish. CRISPR-mediated indels are highlighted in red.

## 1.2 Supplementary Table

| Gene | NCBI ID | sgRNA template | Genotyping primers | |
|---|---|---|---|---|
| | | | Fwd | Rev |
| *cnga2a* | NM_001044746.1 | GACGATGTAACCTAAATCTGTGG | AGCTACTTTCATACTCTACAGT | TCGAACAGGCGTGAGAAGCG |
| | | GTCAAAAGGTGACCTGATGGAGG | GCCAGCTCCAGTCCAGGATGA | ATATCTTCCACCCCTATTCCGT |
| *cnga2b* | XM_021471593.1 | GCGGTTGAAGCGTAGCTGTGGGG | TGGCGTACATCATCACAGGGA | AGTTGGTTCTGGTCTCTGTTCG |
| | | GATGAAGGCATCGCACAGGGAGG | TGGCGTACATCATCACAGGGA | CCAATGCCTGTATGACACAGT |
| *oxt* | NM_178291.2 | GTCTGGAGGTCTGCTGTCCGCGG | AGACACAAACACTAAGTAAG | AGCAGACGGACAGCAGACAC |

**Supplementary Table 1. List of primers used in this study**
